# Increasing COVID-19 Immunization Rates through a Vaccination Program for Hospitalized Children

**DOI:** 10.1097/pq9.0000000000000704

**Published:** 2023-12-05

**Authors:** Victoria Mattick, Katelyn Cappotelli Nevin, Anne Fallon, Stephanie Northwood Darrow, Suzanne Ramazani, Travis Dick, Tina Sosa

**Affiliations:** From the * Pediatric Residency Program, Rochester, N.Y.; † Division of Pediatric Hospital Medicine, Rochester, N.Y.; ‡ Department of Pediatrics, University of Rochester School of Medicine and Dentistry, Rochester, N.Y.; § Operation Excellence, Rochester, N.Y.; ¶ Department of Pharmacy, Rochester, N.Y.

## Abstract

**Introduction:**

Inpatient coronavirus disease 2019 (COVID-19) vaccination initiatives offer a novel strategy to eliminate barriers to care, provide access to interprofessional teams, and decrease COVID-19 morbidity and mortality. Our inpatient vaccination initiative aimed to triple the baseline rate of eligible hospitalized children vaccinated against COVID-19 from 0.95% to 2.85% from December 2021 to June 2022.

**Methods:**

We implemented a COVID-19 vaccination program for pediatric inpatients eligible to receive a dose based on age, current guidelines, and prior doses received. Key drivers included immunization counseling training, identification of eligible patients, and a streamlined workflow. The outcome measure was the percentage of eligible patients who received a vaccine dose during hospitalization. The process measures included the percentage of age-eligible patients who were appropriately screened for prior doses on admission. We designed a clinical decision support system to enhance eligibility identification. The team performed a health equity analysis which stratified patients by social vulnerability index.

**Results:**

During the study period, the average percentage of eligible hospitalized patients vaccinated increased from 0.9% to 3.5%, representing special cause variation and a centerline shift. The average percentage of age-eligible patients screened for prior vaccine doses on admission increased from 66.5% to 81.5%. Patients were more likely to be vaccinated if their clinician was exposed to the clinical decision support system (*P* < 0.01). The social vulnerability index analysis showed no significant differences.

**Conclusions:**

This COVID-19 vaccination initiative highlights how an interprofessional approach can increase vaccination rates in hospitalized children; however, overall inpatient COVID-19 vaccination rates in this setting remained low.

## INTRODUCTION

As of early September 2022, nearly 15 million children in the United States have been diagnosed with coronavirus disease 2019 (COVID-19), representing just under 20% of all cases.^1^ Although children generally experience milder illness than adults, chronic medical problems and very young age are risk factors that predispose children to serious illness, hospitalization, and mortality from COVID-19.^2^ Children are also at risk for unique post-COVID-19 complications, including multisystem inflammatory syndrome in children.^3^ Following clinical trials, mRNA vaccinations proved to be safe and effective in protecting children from severe COVID-19 infections and preventing hospitalization and death.^4–7^ Furthermore, vaccination significantly reduced the rate of multisystem inflammatory syndrome in children in adolescents.^6^

Given the safety and efficacy of COVID-19 vaccinations, providing primary prevention to all eligible individuals is a top healthcare priority. The inpatient setting represents a unique opportunity to provide vaccination, address misinformation, and identify patients at risk for severe COVID-19. Prior studies evaluating the implementation of inpatient influenza vaccination programs in children’s hospitals demonstrated several advantages of inpatient immunization.^8–10^ These included reaching populations without outpatient medical homes, increased access for those with transportation or financial constraints, and vaccine delivery to populations at risk of adverse outcomes. Other advantages included access to an interprofessional care team to educate patients and families, opportunities for coordination with subspecialists, and access to a robust inpatient pharmacy network for vaccine storage and delivery.^8^ Despite these advantages, it is imperative that inpatient vaccination programs effectively address previously identified barriers to vaccination in this setting, including accurately identifying and targeting eligible patients as well as empowering inpatient clinicians to address vaccine hesitancy.^11^

The Food and Drug Administration granted emergency use authorization (EUA) for the Pfizer-BioNTech COVID-19 vaccination for individuals 16 and older in December 2020. This was amended to include adolescents 12–15 years of age in May 2021 and expanded to children ages 5–11 years of age in October 2021.^12^ At that time, our institution did not have a systematic means to provide these vaccinations to hospitalized children. Based on prior studies of inpatient pediatric influenza programs demonstrating 4-fold increases in baseline vaccination rates,^9,13^ we aimed to triple the baseline rate of eligible hospitalized children vaccinated against COVID-19 from 0.95% to 2.85% from December 2021 to June 2022.

## METHODS

### Setting and Context

Our institution is an academic, tertiary care children’s hospital within a hospital that accommodates approximately 6,000 inpatient admissions annually. Inpatient medications, including vaccinations, are processed through a centralized pharmacy within the larger medical center. The institution utilizes an electronic health record (EHR) (Epic).

In September 2021, an interprofessional team of hospital medicine, critical care, and infectious disease attending physicians, pediatric residents, advanced practice providers (APPs), pharmacists, nurses, quality improvement specialists, and clinical informaticists convened to develop and implement an inpatient COVID-19 vaccination program for hospitalized children.

### Ethical Considerations

This study was deemed non-human subjects research by our institution’s subjects review board.

### Interventions

#### Program Development

**Fig. 1.**
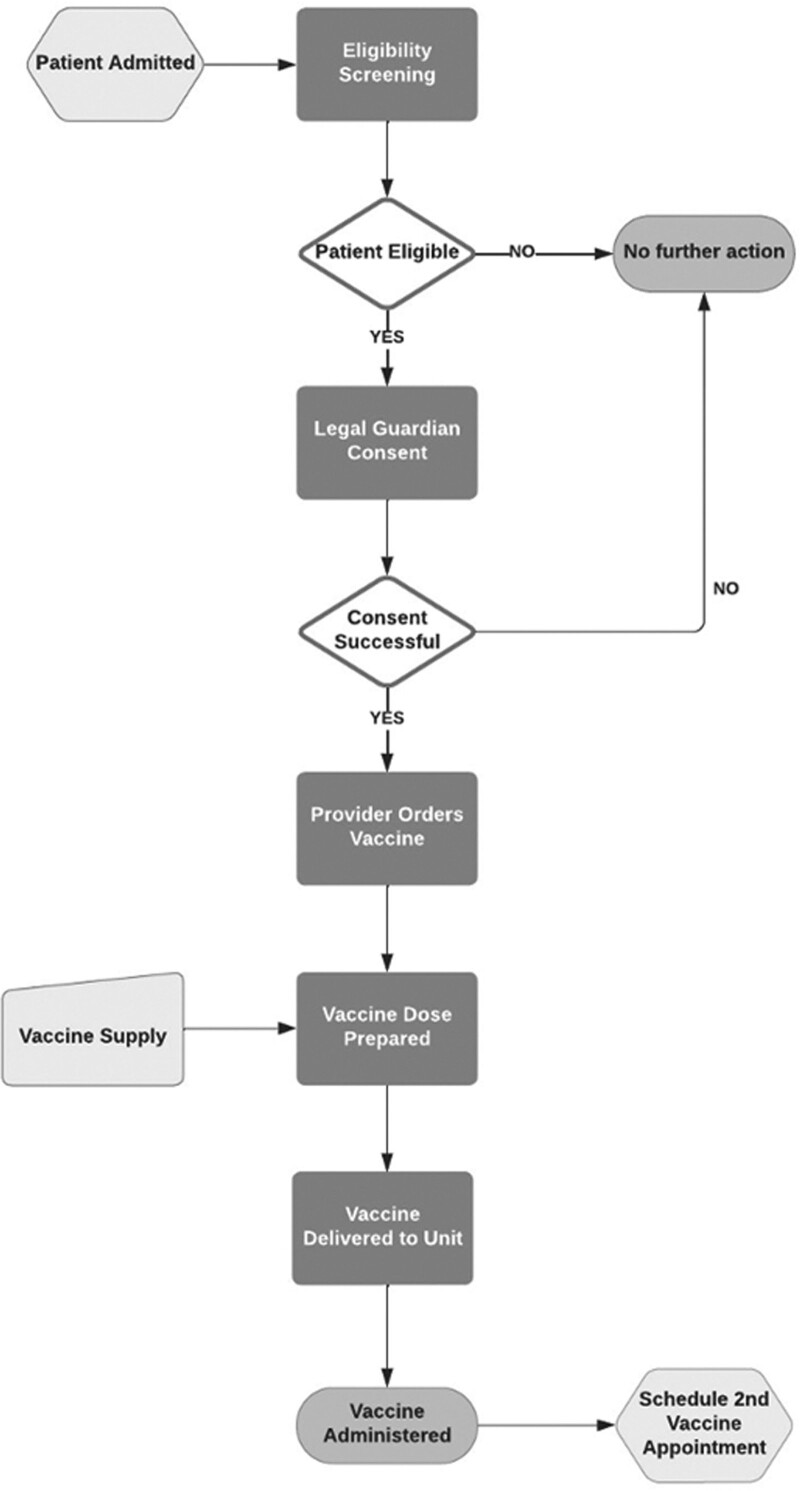
Process map for the inpatient COVID-19 vaccination program.

**Fig. 2.**
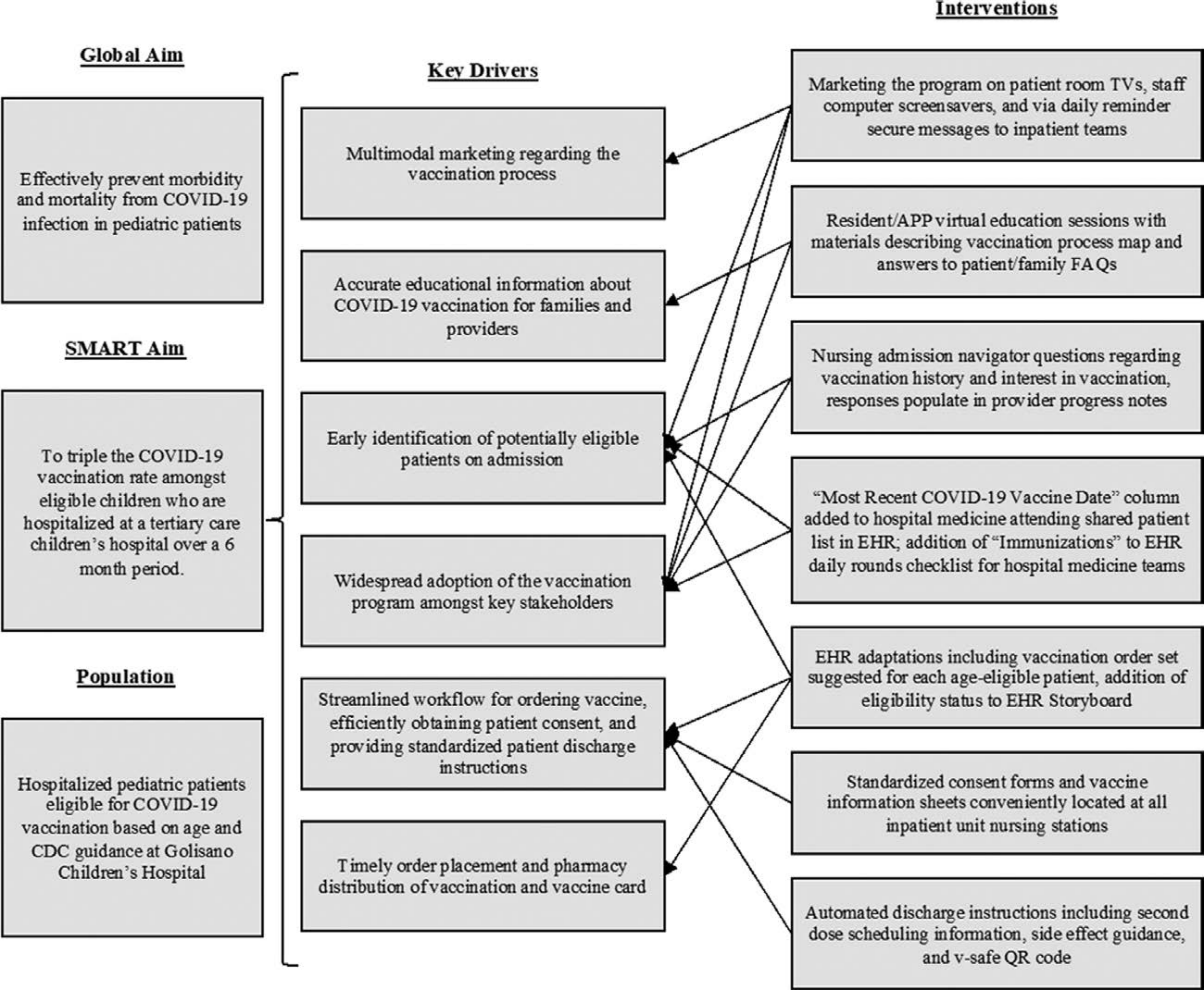
Key driver diagram for COVID-19 vaccination program implementation in the inpatient setting.

Our team used the Model for Improvement^14^ and developed a process map for the COVID-19 inpatient vaccination program (Fig. 1). The process map incorporated expert consensus from the interprofessional team and the limited existing adult literature on an inpatient COVID-19 vaccination program.^15^ We constructed a key driver diagram to describe the theory of improvement for successful implementation and utilization (Fig. 2). Interventions were implemented immediately or tested over time via plan-do-study-act (PDSA) cycles to improve vaccination rates.

As the first step in the process, bedside nurses screened patients for COVID-19 vaccine eligibility by incorporating questions into the existing EHR nursing admission navigator [**Figure 1A, Supplemental Digital Content 1,** which describes vaccination process EHR (Epic) tools. (A) Admission navigator screening questions. (B) Progress note template CDSS. All images © 2021 Epic Systems Corporation, http://links.lww.com/PQ9/A526). These questions asked all patients eligible by age per the Centers for Disease Control (CDC) guidelines about prior doses of the COVID-19 vaccine administered and interest in receiving a vaccine dose before discharge. The answer to the second question auto-populated into the covering clinician’s daily progress note along with a brief review of the steps required by the clinician to proceed with vaccination if the patient and family consented (**Figure 1B, Supplemental Digital Content 1,** http://links.lww.com/PQ9/A526). This clinical decision support system (CDSS) ensured the clinician was prepared with the family’s response when approaching patients regarding vaccination. Those who initially denied interest received additional counseling, and reasons for their hesitancy were addressed by the inpatient team. Although alternative influenza immunization program designs have been described in prior studies, such as standing orders for nurses and automatic vaccination prompts in admission order sets, this COVID-19 vaccination program necessitated clinician notification, shared decision-making with families and consulting services, and inclusion in daily workflows for frequent re-evaluation rather than solely on admission or discharge.^16,17^ This was due to the unique and heightened hesitancy surrounding COVID-19 vaccination for hospitalized children and followed extensive discussion amongst key stakeholders. The dynamic design approach was ultimately selected to best facilitate consistent, daily reminders to clinicians about inpatient vaccine eligibility and preferences to promote effective daily interactions with admitted patients.

Once a family agreed to inpatient vaccination, the primary care team obtained written legal guardian consent using the institution’s standard COVID-19 immunization consent form. Consent forms, available in English and Spanish, were located at nursing stations in each unit alongside the corresponding EUA documents. Providers ordered the vaccine using a standardized order set for all age-eligible children (**Figure 2A and B, Supplemental Digital Content 1**, which describes vaccination process EHR (Epic) order set. (A) Order set main screen. (B) Additional order set components. All images © 2021 Epic Systems Corporation, http://links.lww.com/PQ9/A526). Finally, the ordering clinician was required to certify that the child’s primary service attending and all consulting services were aware of and agreed that vaccination was medically appropriate. This was implemented to ensure that potential vaccine side effects did not interfere with clinical decision-making regarding the patient’s indication for hospitalization.

The interprofessional team decided upon a standard administration time of 6 pm to batch vaccine administrations and minimize waste. The pharmacy department coordinated extra doses from ambulatory vaccine clinics in the evening and ensured those vaccine doses were delivered to the bedside for inpatient orders. The patient’s nurse then administered the vaccination with documentation in the EHR and on the vaccine card provided to the family.

**Fig. 3.**
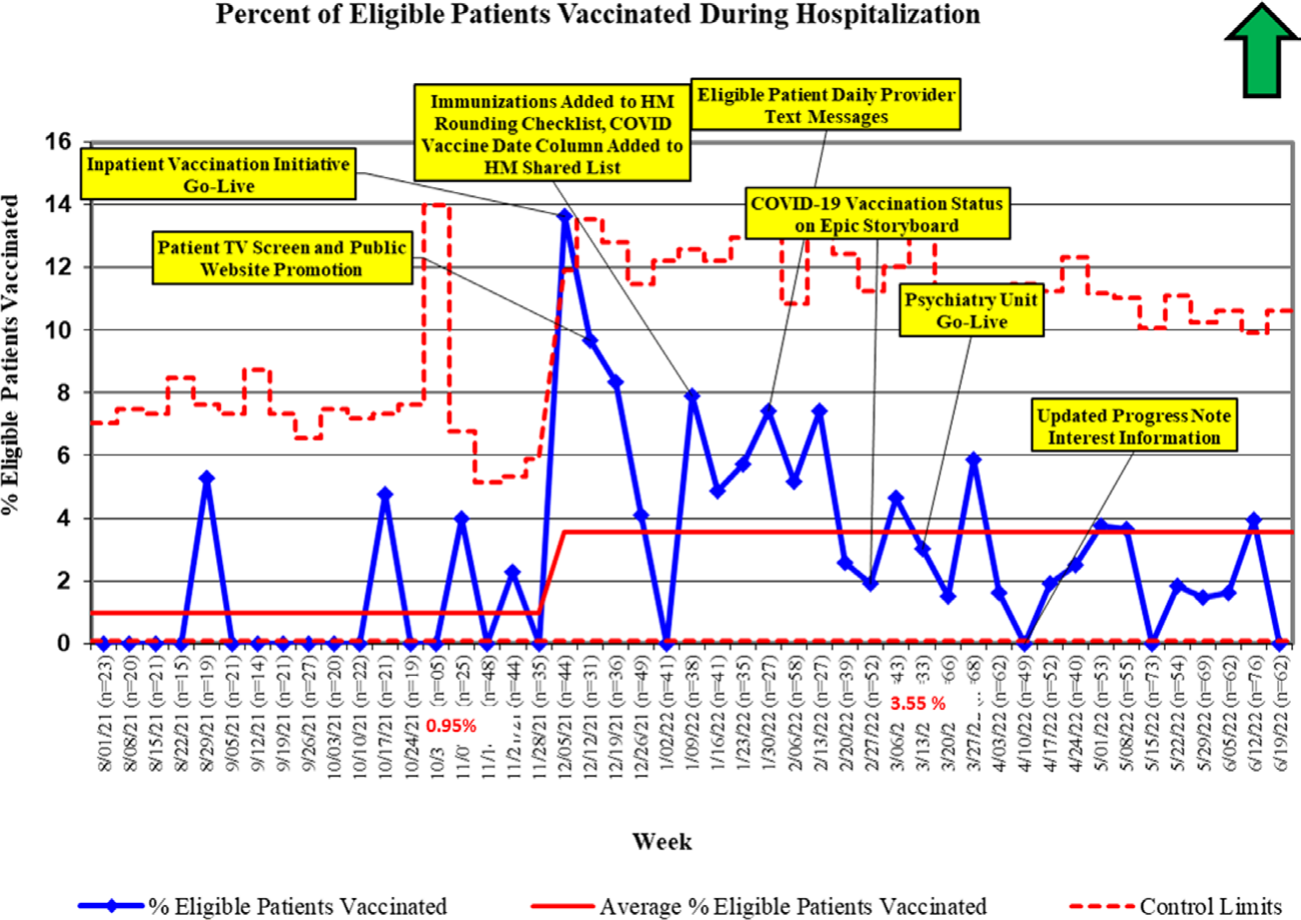
Outcome measure: percent of eligible hospitalized patients vaccinated before discharge. Special cause variation was observed on the statistical process control *P*-chart.

Additionally, standardized COVID-19 vaccination discharge instructions were developed and incorporated into an EHR note template, including when and where to schedule second vaccine doses and anticipatory guidance for side effects and side effect reporting (**Figure 3, Supplemental Digital Content 1**, which describes COVID-19 vaccination discharge instructions, http://links.lww.com/PQ9/A526). If possible, the primary team discussed prescheduling the second vaccine dose with patients and care coordinators before discharge. As a PDSA adaptation, these instructions were later automated to appear in the after-visit summary for any patient with a documented COVID-19 vaccination in the medication administration record, substantially increasing this intervention’s reliability.

#### Staff Education

Staff education regarding the COVID-19 vaccine program began in November 2021, with presentations at several institutional leadership, nursing, residency, APP, and attending stakeholder meetings. Sessions included introducing clinicians to the proposed process map, discussing vaccine counseling, and answering frequently asked questions from families regarding vaccine safety and efficacy in children.

#### Program Marketing

Marketing and promotion of the inpatient vaccination program for staff occurred in several ways. Longitudinally, the program was strongly promoted by institutional leadership, who incorporated it as a standing agenda item on a weekly institution-wide COVID-19 Update Meeting. This provided a platform to inform staff about updated PDSA cycles, review data and progress toward the aim, and obtain feedback. Program marketing towards staff was also prioritized in developing PDSA interventions. First, the team created a promotional screensaver displayed on workstation computers to spread awareness. Next, daily reminder messages were sent to the inpatient clinicians via a secure internal messaging platform; these were later individualized to the covering clinicians for patients identified as eligible on a daily admitted patient query.

The first program marketing intervention directed towards patients and families was the addition of inpatient COVID-19 vaccination opportunity information to the GetWellNetwork as an automatic pop-up that appeared at a standard time on the second day of admission on the patient room television screen. Simultaneously, the program launch was promoted on the institutional website.

#### Pediatric Hospital Medicine Service-targeted Interventions

Because most eligible patients were admitted to the hospital medicine service, two interventions targeted this service line. A column was added to the shared attending patient list in the EHR with the patient’s last documented COVID-19 vaccine administration date. Building upon previous quality improvement work at our institution that standardized a rounding checklist for attending physicians,^18^ the “LEAD” acronym (intravenous Lines, Expiring orders, Antimicrobials, and Diagnostic testing) was adapted to “I LEAD,” adding “Immunizations” with a specific reference to COVID-19 and influenza vaccines for eligible patients. This checklist auto-populated into a templated attending progress note attestation.

#### Epic Storyboard Enhancement

**Fig. 4.**
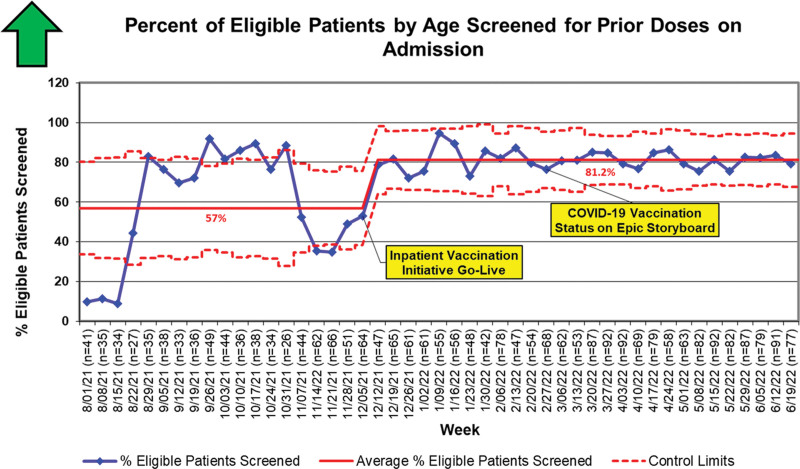
Process measure: percent of age-eligible patients screened for prior vaccine doses on admission. Special cause variation was observed on the statistical process control *P*-chart.

An additional CDSS was developed and incorporated into the Epic Storyboard (a vertical sidebar visible throughout all areas of the patient chart that features key demographic and clinical information) to enhance the visibility of the patient’s eligibility for a vaccine dose (**Figure 4, Supplemental Digital Content 1**, which describes Epic Storyboard eligibility CDSS. Image © 2022 Epic Systems Corporation, http://links.lww.com/PQ9/A526). Utilizing the Immunization History section of the EHR, the patient’s eligibility for COVID-19 vaccination would appear on the main dashboard of their electronic chart. By hovering over or selecting this feature, additional information regarding the timing of prior doses and eligibility was available.

### Study of the Interventions

EHR data including nursing admission screening questions, immunization history, patient demographic data, and the medication administration record were extracted weekly via an Epic Reporting Workbench report. Monthly queries from the EHR databases were created to obtain data on length of stay, healthcare reutilization (including readmission and emergency department or urgent care visits within 7 days of discharge), social vulnerability index (SVI), and exposure to the CDSS in the progress note template.^19^ To ensure vaccination opportunities were addressed across all subspecialty services and hospital units, Pareto charts of missed opportunities by subspecialty were created at multiple points during the study period to inform targeted messaging to leadership to promote vaccination. After hospital discharge, a small group of team members conducted follow-up calls to obtain family feedback and inform future interventions.

### Measures

The primary outcome measure was the percentage of eligible patients vaccinated before hospital discharge. The operational definition of an eligible patient included a hospitalized child eligible to receive a dose (primary series or booster) of the COVID-19 vaccine based on age and prior doses received per current CDC recommendations. The inclusion criteria were modified over time as eligibility expanded. The process measures included the percentage of age-eligible patients with a response documented for the nursing admission navigator screening questions (“yes” or “no,” rather than “unknown” or blank) and the number of patients who interacted with the GetWellNetwork, an automatic pop-up that appeared at a standard time on the second day of admission on the patient room interactive television screen. Balancing measures included hospital length of stay and healthcare reutilization following discharge.

### Analysis

Outcome and process metrics were tracked via prospective time series analyses on statistical process control charts and analyzed utilizing established rules for special cause variation.^20,21^ Balancing measures were tracked on run charts. To assess for equitable screening of hospitalized patients regarding prior vaccine doses and interest in receiving a vaccination, patients were stratified into quartiles (high, medium-high, medium-low, and low social vulnerability) using the CDC’s SVI for a health equity analysis, and these quartiles were compared via Chi-square test.^19^ Although the progress note template CDSS was incorporated into the most commonly utilized progress note template in our EHR, we recognized that some clinicians utilized individualized progress note templates and, therefore, would not be exposed to this CDSS. This provided an opportunity to evaluate the effectiveness of this intervention. A retrospective analysis was performed to evaluate the effectiveness of the progress note template CDSS tool in which subgroups were compared using Fisher’s exact test.

## RESULTS

For the primary outcome measure, the average percentage of eligible hospitalized patients vaccinated increased from 0.95% (4/420) to 3.55% (51/1435) during the study period, representing special cause variation and a centerline shift on a statistical process control *P*-chart (10/11 consecutive points above the previous centerline—American Society of Quality Engineers). This represents a nearly quadrupled vaccination rate, with 51 inpatients vaccinated and an absolute vaccine increase of 37 administered over the mean baseline rate (Fig. 3).

Concerning the process measures, the average percentage of age-eligible patients screened for prior vaccine doses on admission increased from 57.3% (418/729) to 81.2% (1,557/1,918) with a special cause centerline shift (eight points in a row) observed on a *P*-chart (Fig. 4). Notably, among patients who were not screened, only 1.5% (10/674) of eligible patients were vaccinated during the hospitalization, and no special cause variation was observed in nonscreened patients’ vaccination rates over time. The percentage of patients who interacted with the automatic pop-up on the GetWellNetwork television screen during their admission was 81% during the study period. Both balancing measures demonstrated only common cause variation, with the median length of stay for eligible patients remaining at 4.27 days and the median readmission rate stable at 2.17% throughout the study period.

There was no significant difference in the percentage of age-eligible patients who asked vaccine screening questions when data was stratified by the SVI quartile (*P* = 0.80). Although the sample size was limited, SVI analysis was also performed for vaccination status. There was no significant difference in the percentage of age-eligible patients vaccinated when SVI quartiles were compared (*P* = 0.50, Table 1).

**Table 1.** Health Equity Analysis^*^

Social Vulnerability Index Category	Eligible Inpatients Vaccinated, N (%)
High social vulnerability, n = 610	14 (2)
Med-high social vulnerability, n = 548	18 (3)
Med-low social vulnerability, n = 500	13 (3)
Low social vulnerability, n = 495	9 (2)

^*^Patients were grouped into quartiles (high, med-high, med-low, and low) using the CDC’s SVI framework. There was no statistically significant difference between the percentage of eligible inpatients vaccinated by quartile (*P* = 0.50).

For the progress note CDSS effectiveness analysis, of 746 total eligible patients, 57.0% (425/746) of progress notes were written by physicians utilizing the embedded CDSS based on the admission screening questions, and 43.0% (321/746) of progress notes were individualized and did not contain the CDSS. Patients whose clinicians were exposed to the progress note CDSS were significantly more likely to be vaccinated [vaccination rate of 6.1% (26/425)] compared to those patients whose clinicians were not exposed [vaccination rate of 1.9% (6/321)] (*P* < 0.01).

Postdischarge calls to vaccinated patients and families were conducted to inquire about vaccine side effects if second doses were received after hospitalization, if the inpatient team assisted with scheduling the second dose, and suggestions for improvement in the vaccination process. These calls revealed that families were satisfied with the vaccination program, and no significant concerns/suggestions emerged. In some cases, the study team was able to assist families in scheduling subsequent vaccine doses if indicated.

## DISCUSSION

Through an interprofessional and multimodal approach that leveraged education, communication, EHR enhancements, and clinical decision support, implementing a systematic COVID-19 vaccination program was associated with a nearly quadrupled vaccination rate for eligible hospitalized pediatric patients at a tertiary care children’s hospital. Specifically, our initiative demonstrates how to quickly launch an inpatient vaccination program in the setting of a global pandemic and a novel vaccination with rapidly evolving eligibility criteria. This was completed while the healthcare system faced ongoing challenges (eg, staffing, supply chain, new COVID-19 variants) related to the pandemic. Our data demonstrate that eligible patients screened through this program and exposed to our interventions were over twice as likely to be vaccinated in the hospital setting as opposed to those not screened (3.55% versus 1.5%), which provides evidence to associate our interventions with the observed improvement. Although this COVID-19 vaccination program was associated with a significant relative increase in vaccinations received, challenges related to inpatient COVID-19 vaccination were faced, and overall vaccination rates among eligible pediatric inpatients were low. This underscores the importance of future studies to elucidate specific barriers to vaccination in the inpatient setting, which can serve high-risk populations and overcome significant outpatient challenges (eg, transportation). As seen in Figure 3, special cause variation was immediately achieved with the launch of the vaccination program in early December 2021. Although an overall increased mean vaccination rate was sustained, the number of vaccinations administered waned over time. This trend is consistent with findings in adult inpatient COVID-19 vaccination program studies.^15^ Potential causes for this decrease included the enhanced opportunity to vaccinate early adopters in inpatient and outpatient settings following eligibility expansions, thus leading to increases in both a vaccine-hesitant population and an already vaccinated population encountered in the inpatient setting over time. Additionally, vaccine hesitancy increased as the age of eligibility decreased. Furthermore, enthusiasm for vaccination was likely higher during periods of greater COVID-19 case numbers, including during the Omicron variant surge in the late fall and early winter of 2021–2022.

To maintain vaccination rates despite these challenges, progressing from lower reliability to higher reliability interventions was critical to sustain a vaccination rate above baseline. Early education and marketing proved to be intensive and less effective as vaccines became more readily available, reinforcing the findings of multiple COVID-19 vaccination studies in the outpatient setting.^22,23^ Thus, higher reliability interventions such as the CDSS and the addition of the I LEAD acronym were implemented to ensure sustainability. Adding these interventions to the note template ensured that the hospitalist team addressed COVID-19 vaccination daily despite the season, variant surge, and patient population. Incorporating these changes into a means already embedded into the daily workflow further strived to eliminate the time-intensive efforts that exist with COVID-19 vaccination while maximizing impact.

### Limitations

This inpatient COVID-19 vaccination program was initiated at a tertiary care children’s hospital, with resources including an established EHR, informatics and quality improvement specialists, residents, APPs, attending physicians, and pediatric pharmacists able to support its implementation. This significant multidisciplinary effort may limit the generalizability to other centers.

Unlike inpatient influenza vaccination programs, additional system-based challenges specific to COVID-19 vaccination included limited shelf life, navigating state-managed supply and strategic conservation to avoid wasted doses, defining and raising awareness for eligible patients, and the necessity for subsequent doses after discharge.^8^ Potential clinician-related factors contributing to low vaccination rates included clinician hesitancy based on available data, logistic barriers including competing priorities, time constraints, and the clinician perception that inpatient providers may lack the skills and longitudinal relationship with families to discuss vaccination, especially in vaccine-hesitant encounters.^11^ Both system- and clinician-related factors were significantly exacerbated by ongoing staffing shortages related to the pandemic. Patient-related challenges, which span multiple types of vaccines, included lack of rapport or perceived judgment based on vaccine status, not desiring vaccination during an acute illness, preference to receive the vaccine outpatient with the patient’s primary care provider, beliefs against vaccination, and lack of trust in the medical system.^11,15,24^ Specific to the COVID-19 vaccine, studies surveying parental opinions about the vaccine have shown lack of confidence in vaccine efficacy and safety, lack of trust in the government, and perceptions that children are not susceptible to COVID-19 as consistent factors influencing vaccine hesitancy.^25^

In addition to the above challenges faced by our improvement team, significant limitations on vaccination orders and delivery times within our pharmacy department provided further challenges. The pharmacy could accommodate exceptions with proactive outreach by the primary teams, but consolidation of the vaccine delivery time from only 4 to 6 pm likely precluded multiple patients from vaccination.

### Conclusions

Implementing an inpatient COVID-19 vaccination program at a tertiary care children’s hospital was associated with increased COVID-19 vaccination rates among eligible patients. Successful implementation of this program required interprofessional communication, logistical flexibility, reliance on electronic medical record technology, and a team-based approach. Additionally, frequent analysis of real-time data and reflection on program challenges were necessary to develop innovative solutions and ensure program sustainability. Despite these interventions, overall COVID-19 vaccination rates remained low, with the number of vaccinations administered weaning over time. Further studies investigating specific patient and family, provider, and systems-based barriers to inpatient pediatric COVID-19 vaccination are needed to understand this trend and inform areas for high-impact, targeted interventions to increase COVID-19 vaccination rates in this population.

## DISCLOSURE

The authors have no financial interest to declare in relation to the content of this article.

## Supplementary Material


