# Supplementary material for: Increasing COVID-19 Immunization Rates through a Vaccination Program for Hospitalized Children
Source: Pediatr Qual Saf. 2023 Dec 5;8(6):e704. doi: 10.1097/pq9.0000000000000704 (PMC10697599; doi:10.1097/pq9.0000000000000704)

SDC, Figure 1. *Vaccination Process Electronic Health Record (Epic) Tools.* A) Admission Navigator Screening Questions. B) Progress Note Template CDSS. All images © 2021 Epic Systems Corporation.

1A.

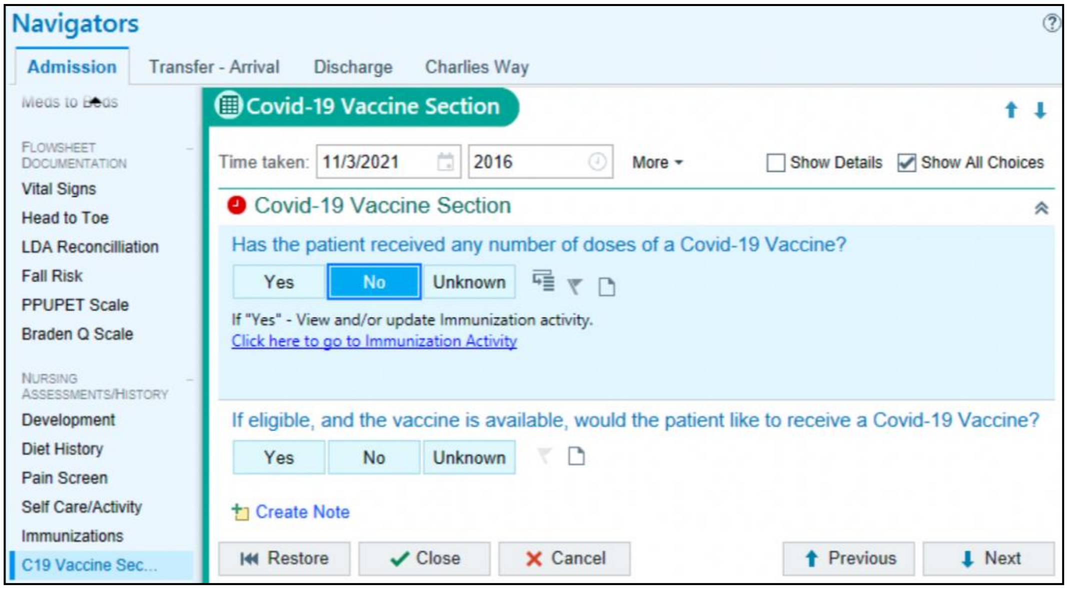

1B.

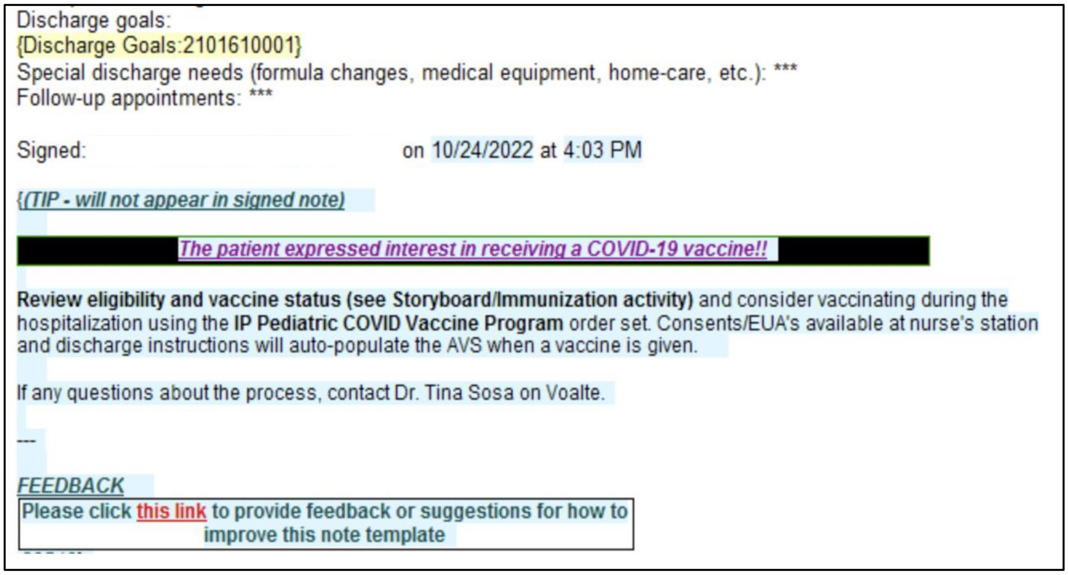

SDC, Figure 2. *Vaccination Process Electronic Health Record (Epic) Order Set.* A) Order Set Main Screen. B) Additional Order Set Components. All images © 2021 Epic Systems Corporation.

2A.

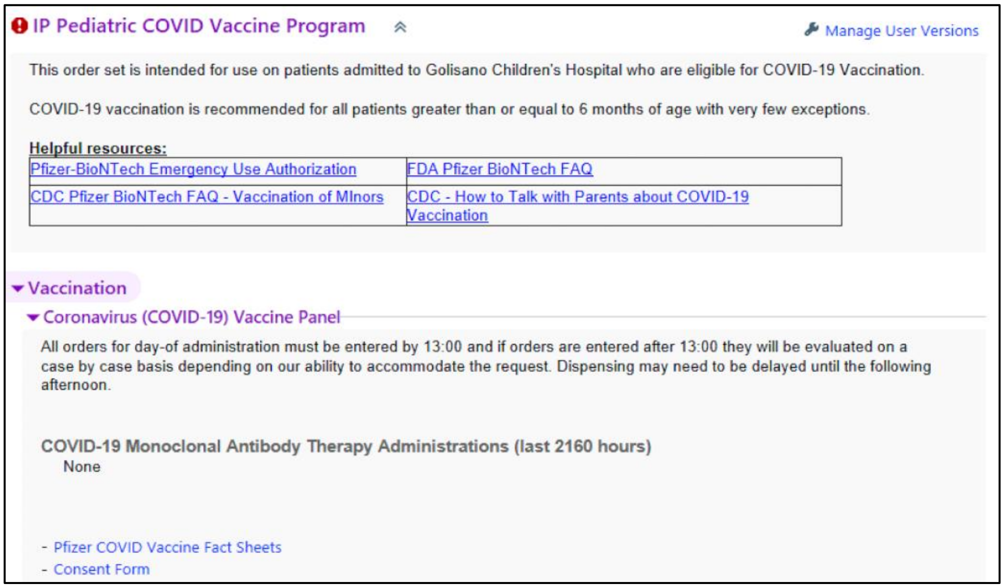

2B.

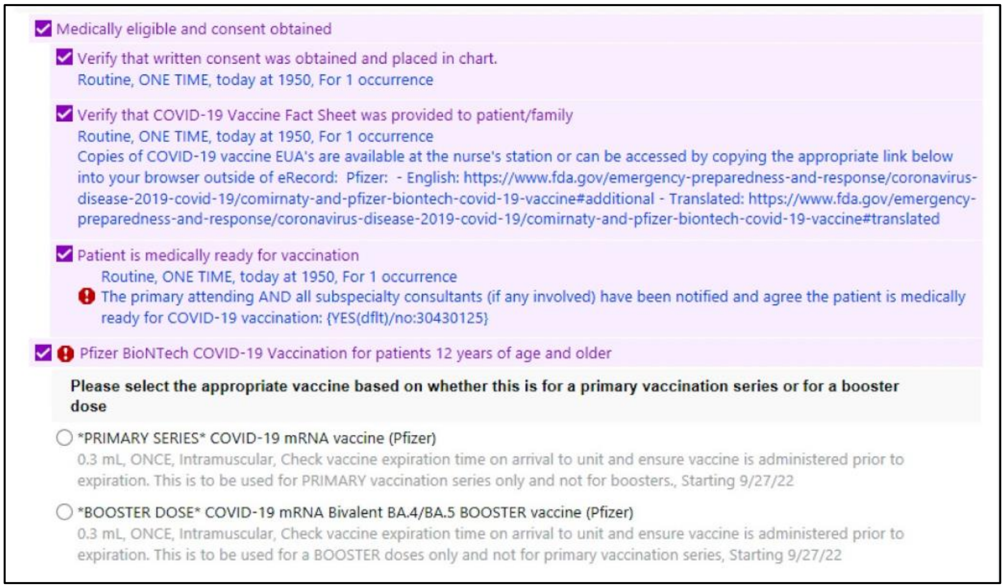

SDC, Figure 3. *COVID-19 Vaccination Discharge Instructions.*

COVID-19 Vaccine Discharge Instructions

You received a Pfizer-BioNTech COVID-19 vaccination during your admission.

If you experience the following mild symptoms within the first 48 hours after vaccination, these could be side effects from the vaccine:

Fever or Chills   Tiredness
Headache   Muscle or joint pain

For relief from mild symptoms, you can safely use over-the-counter medicines (such as Acetaminophen (Tylenol)).

If these symptoms last longer than 48 hours, consider discussing the symptoms with your doctor.

Occasionally, there can be more serious side effects. If you experience any of the following symptoms, **these may be signs of a severe allergic reaction and you should call 9-1-1 or go to the nearest hospital**:

- Difficulty breathing
- Swelling of the face and throat
- Rash all over the body

We recommend V-Safe – a free, easy-to-use, and confidential smartphone-based tool. It uses text messaging and web surveys to provide personalized health check-ins after you receive a COVID-19 vaccination. Through V-safe, you can report how you feel after getting vaccinated.

https://www.cdc.gov/coronavirus/2019-ncov/vaccines/safety/v-safe-register-dependents.html

Where can you get additional vaccine doses if they are needed?

1. Pharmacies: Many local pharmacies are offering the COVID-19 vaccine, including Walgreens and CVS. You can check your local pharmacy's website to see if vaccination walk-ins or appointments are available.

2. County Health Departments:

Inside NY State

https://www.nysacho.org/directory/

Outside NY State

https://www.cdc.gov/publichealthgateway/healthdirectories/healthdepartments.html

3. Public Vaccination Clinics: Open vaccination clinics may be available at your local hospitals and healthcare system sites. Here are resources to find more information on these options.

- Vaccines.gov
- Text message your ZIP code to 438829
- Call 1-800-232-0233

SDC, Figure 4. *Epic Storyboard Eligibility Clinical Decision Support System*. Image © 2022 Epic Systems Corporation.

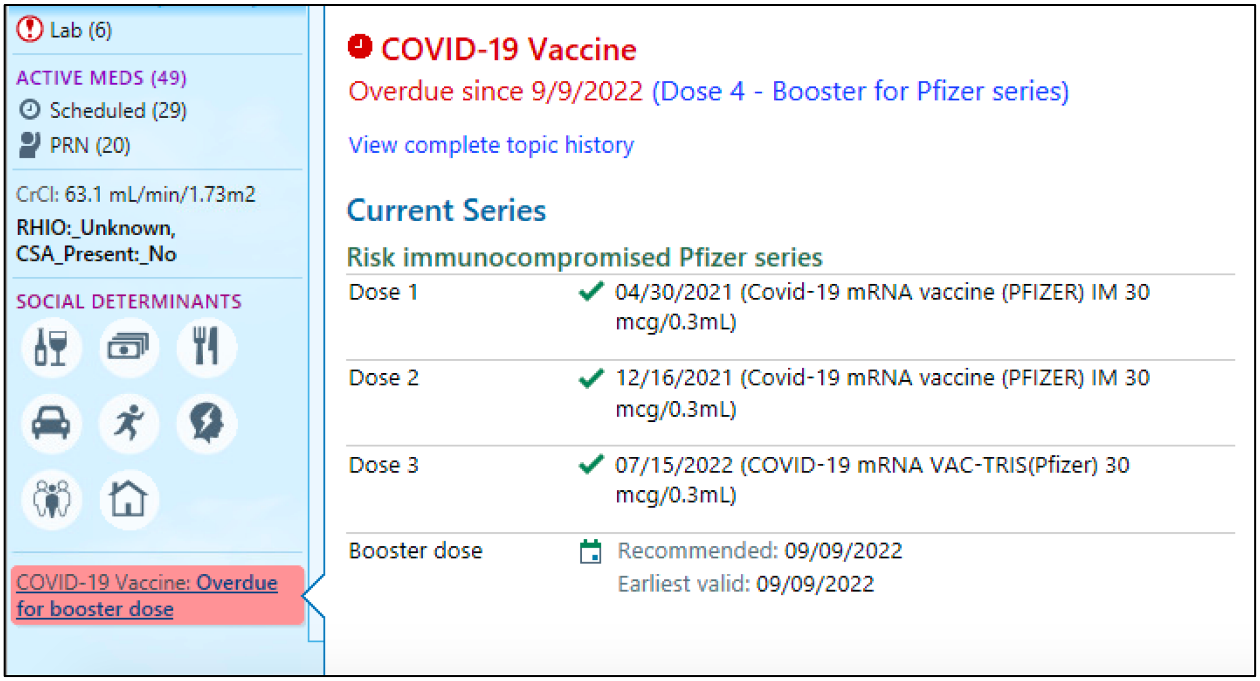

Supplement: Supplementary file 1 [file pqs-8-e704-s001.pdf]
